# Ecology, Epidemiology and Disease Management of *Ralstonia syzygii* in Indonesia

**DOI:** 10.3389/fmicb.2018.00419

**Published:** 2018-03-13

**Authors:** Irda Safni, Siti Subandiyah, Mark Fegan

**Affiliations:** ^1^Department of Agrotechnology, Faculty of Agriculture, Universitas Sumatera Utara, Medan, Indonesia; ^2^Department of Entomology and Plant Pathology, Faculty of Agriculture, Gadjah Mada University, Yogyakarta, Indonesia; ^3^Research Center for Biotechnology, Gadjah Mada University, Yogyakarta, Indonesia; ^4^Agriculture Victoria, Department of Economic Development, Jobs, Transport and Resources, Bundoora, VIC, Australia

**Keywords:** *Ralstonia syzygii*, bacterial wilt, Indonesia, ecology, epidemiology, disease management

## Abstract

*Ralstonia solanacearum* species complex phylotype IV strains, which have been primarily isolated from Indonesia, Australia, Japan, Korea, and Malaysia, have undergone recent taxonomic and nomenclatural changes to be placed in the species *Ralstonia syzygii*. This species contains three subspecies; *Ralstonia syzygii* subsp. *syzygii*, a pathogen causing Sumatra disease of clove trees in Indonesia, *Ralstonia syzygii* subsp. *indonesiensis*, the causal pathogen of bacterial wilt disease on a wide range of host plants, and *Ralstonia syzygii* subsp. *celebesensis*, the causal pathogen of blood disease on *Musa* spp. In Indonesia, these three subspecies have devastated the cultivation of susceptible host plants which have high economic value. Limited knowledge on the ecology and epidemiology of the diseases has hindered the development of effective control strategies. In this review, we provide insights into the ecology, epidemiology and disease control of these three subspecies of *Ralstonia syzygii*.

## Introduction

Indonesian agriculture is dedicated to the production of food crops for local consumption by an ever expanding population (Maulana and Sayaka, 2007), agriculture also plays a significant role in the Indonesian economy (Cervantes-Godoy and Dewbre, 2010). From 2001 to 2008, national spending on agriculture increased from 11 billion rupiah to 53 billion rupiah, which is an average increase of 11% annually (Armas et al., 2012). Plant and animal diseases are primary constraints affecting agricultural production, especially in tropical countries such as Indonesia (Magarey et al., 2010; Prabaningrum and Moekasan, 2014; Drenth and Guest, 2016). Bacterial wilt disease, caused by members of the *Ralstonia solanacearum* species complex, is a serious disease of crop plants in Indonesia. Geddes (1992) ranked bacterial wilt as the 6th most detrimental plant pest and disease in Indonesia after the damage caused by rats (*Ratus* spp.), stem borers (*Scirpophaga innotata, S. incertula*, and *Chilo suppressalis*), bacterial rice blight (*Xanthomonas oryzae* pv. *oryzae*), the brown planthopper (*Nilaparvata lugens*) and the oriental leafworm moth (*Spodoptera litura* and *S. exigua*).

Bacterial wilt disease on *Musa* spp., called blood disease, was first reported on Selayar Island, South Sulawesi (formerly Celebes) in 1906 by Gäumann (1921). The local people named the disease “blood disease” to reflect the reddish brown bacterial exudate secreted from internal vascular tissue of pseudostems and fruits of infected bananas (Gäumann, 1924). Bacterial wilt affecting *Syzygium aromaticum* trees, Sumatra disease or wooden vessel bacteria on *S. aromaticum*, was first observed in Sumatra, Indonesia in 1975 (Waller and Sitepu, 1975).

In Indonesia, bacterial wilt disease occurs on a wide varieties of crops and both blood disease of banana and Sumatra disease of clove have significantly impacted the banana and clove industries, respectively. The impact on banana production due to blood disease was estimated to be approximately 36% in 1991 (Muharam and Subijanto, 1991). In Lampung, Southern Sumatra, losses due to blood disease have been estimated to reach 64% (Cahyaniati et al., 1997). Production of *S. aromaticum* has decreased rapidly since 1996, mainly due to Sumatra disease (Suryana et al., 2004).

## Phylogeny, Classification and Geographic Distribution of *R. syzygii* Subspecies

Members of the *R. solanacearum* species complex have the most diverse host range and widest geographic distribution of any plant pathogenic bacterium (Elphinstone, 2005). The term “species complex” was introduced by Gillings and Fahy (1994) to indicate the high degree of phenotypic and genotypic diversity within the species *R. solanacearum*. Within the *R. solanacearum* species complex, four genetic groups, termed phylotypes, have been defined (Fegan and Prior, 2005; Prior and Fegan, 2005). Phylotypes I, II, and III are composed of strains mainly from Asia, America, and Africa, respectively, while Phylotype IV is primarily composed of strains from Indonesia but also occurs in a number of other Asian countries (Fegan and Prior, 2005).

Prior to the phylotyping classification system, *R. solanacearum* species complex strains were grouped into five races on the basis of host range (Buddenhagen, 1962; He et al., 1983) and five biovars based on the metabolism of three disaccharides (maltose, lactose, and cellobiose) and three hexose alcohols (sorbitol, mannitol, and dulcitol), the production of nitrite from nitrate and the production of gas from nitrate (Hayward, 1964, 1991, 1994a,b). While the biovar concept is applicable to *R. solanacearum* strains the biovar typing system has not been applied to *R. syzygii* or banana blood disease strains. The race and biovar classification system do not relate to each other with the exception that race 3 strains causing brown rot of *Solanum tuberosum* L. are generally considered to be equivalent to biovar 2 (Li et al., 2014).

**FIGURE 1:**
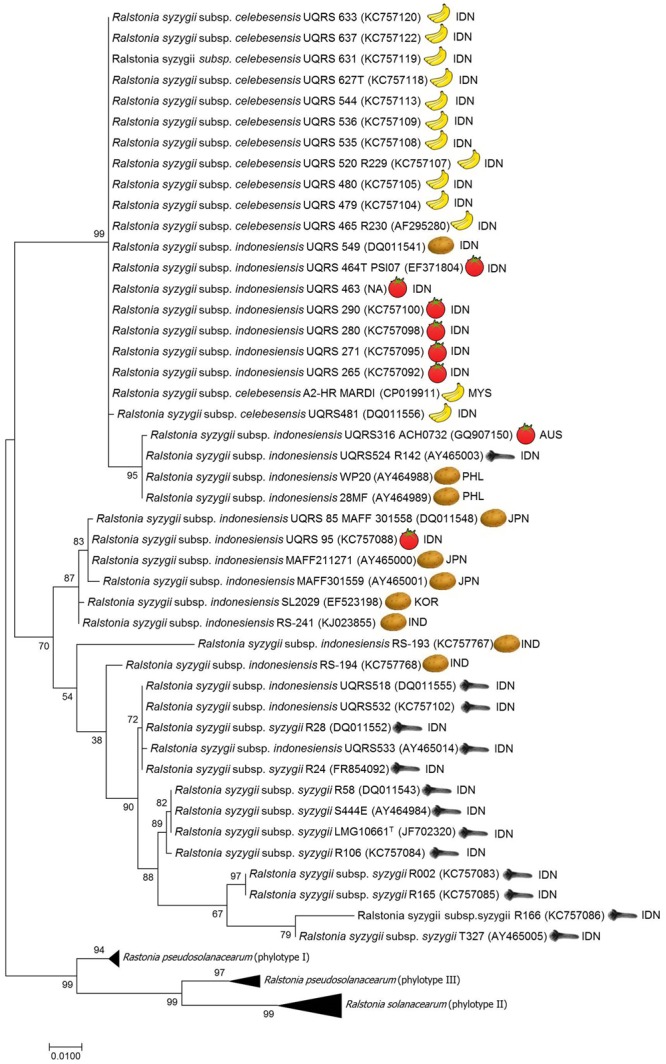
Molecular Phylogenetic analysis of *egl* gene sequences data using the Maximum Likelihood method. The evolutionary history was inferred by using the Maximum Likelihood method based on the Tamura-Nei model. The percentage of trees in which the associated taxa clustered together is shown next to the branches. The tree is drawn to scale, with branch lengths measured in the number of substitutions per site. There were a total of 794 positions in the final dataset. Evolutionary analyses were conducted in MEGA7.0 (Kumar et al., 2016). ISO geographical codes: AUS, Australia; IDN, Indonesia; IND, India; JPN, Japan; KOR, Korea; MYS, Malaysia; PHL, Philippines.

Recently, members of the *R. solanacearum* species complex have undergone a taxonomic revision (Safni et al., 2014). The reclassification of members of the *R. solanacearum* species complex based on a polyphasic study of phenotypic and genotypic characteristics led to the description of three species, *R. solanacearum, R. pseudosolanacearum*, and *R. syzygii* which are comprised of phylotype II, Phylotypes I and III, and phylotype IV strains, respectively (Safni et al., 2014). This classification has been confirmed by further proteomic and genomic characterisation of *R. solanacearum* species complex strains (Prior et al., 2016). Of the three species *R. syzygii*, as defined by Safni et al. (2014), is the most diverse group and contains three subspecies *R. syzygii* subsp. *syzygii, R. syzygii* subsp. *indonesiensis* and *R. syzygii* subsp. *celebesensis*. DNA-DNA Hybridization (DDH) is a molecular approach used to compare the overall similarity of whole genomes among different organisms (Rossello-Mora, 2006). The DDH value is expressed as a percentage homology, which a value of greater than 70% relatedness has been proposed as a recommended standard for species delineation (Wayne et al., 1987). However, a more relaxed boundary value The DDH values among the three subspecies of *R. syzygii* ranges from 67 to 100%, each subspecies can also be differentiated using phenotypic and genotypic characteristics in combination with pathogenicity (Safni et al., 2014). *R. syzygii* subsp. *syzygii* is the pathogen which causes Sumatra disease of clove trees and has only been described to occur in Indonesia. This subspecies, which contains the type strain of the species, was originally described as *Pseudomonas syzygii* by Roberts et al. (1990). This subspecies contains group of strains which are able to utilize only a small number of carbon sources (Roberts et al., 1990; Safni et al., 2014). *R. syzygii* subsp. *celebesensis*, the causal agent of blood disease on banana and plantain, occurs in Indonesia but has also been observed on the island of New Guinea (Davis et al., 2001) and has recently been identified in Malaysia (Kogeethavani et al., 2014; Teng et al., 2016). *R. syzygii* subsp. *celebesensis* strains are more metabolically active than *R. syzygii* subsp. *syzygii* strains but are less metabolically active than *R. syzygii* subsp. *indonesiensis* strains. Strains of *R. syzygii* subsp. *indonesiensis* cause bacterial wilt of a range of solanaceous host plants and have been recorded as being present in Indonesia, Australia, and Japan (Horita et al., 2010; Suga et al., 2013), Korea (Jeong et al., 2007), India (Gurjar et al., 2015) and the Philippines (Villa et al., 2005) (**Figure 1** and **Table 1**).

**Table 1:** Geographic distribution and hosts of *Ralstonia syzygii*.

Species	Country	Host	Biovar	Reference
*R. syzygii* subsp. *syzygii*	Indonesia	*Syzygium aromaticum*	NA^1^	Waller and Sitepu, 1975
		Indigenous species of Myrtaceae	NA	Eden–Green et al., 1992; Lomer et al., 1992
*R. syzygii* subsp. *celebesensis*	Indonesia	Banana and plantain	NA	Gäumann, 1921; Subandiyah et al., 2005
	Malaysia	*Musa* spp.	NA	Kogeethavani et al., 2014; Teng et al., 2016
	The island of New Guinea	*Musa* spp.	NA	Davis et al., 2001
*R. syzygii* subsp. *indonesiensis*	Indonesia	*S. lycopersicum*	2T	This study, Safni et al., 2014
		*Capsicum annuum*	2T	This study, Safni et al., 2014
		*S. tuberosum*	2T	This study, Safni et al., 2014
		*S. aromaticum*	1, 2T	This study, Safni et al., 2014
	Japan	*S. tuberosum*	2T	Horita et al., 2010; Suga et al., 2013
	India	*S. tuberosum*	2T	Gurjar et al., 2015
	Australia	*S. lycopersicum*	2	Fegan et al., 1998a,b
	Philippines	*S. tuberosum*	2T	Villa et al., 2005
	Korea	*S. tuberosum*	2	Jeong et al., 2007
		*S. lycopersicum*	2	Jeong et al., 2007

*^1^Not applicable. S. tuberosum is Solanum tuberosum.*

Phylogenetic analysis of endoglucanase (*egl*) gene sequence data has been used to establish the evolutionary history of the *R. solanacearum* species complex and to determine the phylotype to which a strain belongs (Villa et al., 2005; Fegan and Prior, 2006). Using phylogenetic analysis of *egl* gene sequence data on *R. syzygii* strains reveals that the genetic diversity of these three subspecies varies (see **Figure 1**). All strains of *R. syzygii subsp. celebesensis* cluster together into a monophyletic group with certain *R. syzygii* subsp. *indonesiensis* strains from Indonesia. In contrast, strains of *R. syzygii* subsp. *syzygii* exhibit a greater degree of diversity in *egl* sequences but the largest level of genetic diversity in the species is present in strains belonging to *R. syzygii* subsp. *indonesiensis*. Analysis of *egl* gene sequence data using different phylogenetic methods, including Maximum likelihood, Neighbor joining, unweighted pair group method (UPGMA), and minimum evolution, produce congruent phylogenies (results not shown) but are not able to resolve the three subspecies with certain strains of *R. syzygii* subsp. *indonesiensis* being closely related to *R. syzygii subsp. celebesensis* and others more closely related to *R. syzygii* subsp. *syzygii* (Safni, 2014) (**Figure 1**). By employing multilocus sequence analysis (MLSA) Wicker et al. (2012) were able to identify eight groups, or clades, among the four phylotypes. Although only a few strains of *R. syzygii* were examined by MLSA results similar to the analysis of *egl* sequence data were found. MLSA was not able to clearly delineate the three subspecies of *R. syzygii* and certain *R. syzygii* subsp. *indonesiensis* strains from Indonesia clustered with *R. syzygii subsp. celebesensis* or *R. syzygii* subsp. *syzygii* strains (Wicker et al., 2012). It is interesting to note that the *R. syzygii* subsp. *indonesiensis* strains clustering closely with *R. syzygii* subsp. *syzygii* are also isolated from clove trees.

While a low level of genetic diversity has been described between *R. syzygii* subsp. *celebesensis* strains by sequence analysis of *egl* genes (Fegan and Prior, 2006; Safni et al., 2014) (**Figure 1**) and the 16S–23S intergenic spacer (ITS) region (Safni et al., 2014) and also genomic DNA fingerprinting patterns by rep-PCR and random amplified polymorphic DNA analysis (Thwaites et al., 1999), pulsed-field gel electrophoresis has revealed a degree of diversity within strains of the pathogen (Hadiwiyono et al., 2011).

## Ecology and Epidemiology

### Sumatra Disease of Clove Trees: *R. syzygii* subsp. *syzygii*

**FIGURE 2:**
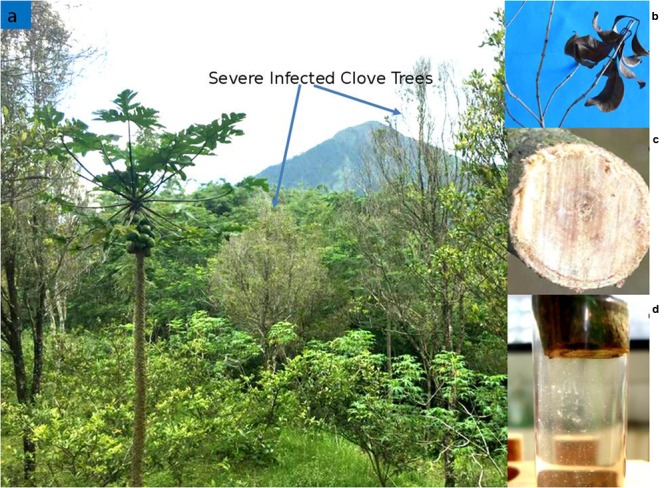
**(a)** Field infection of Sumatra Disease of Clove caused by *Ralstonia syzygii* subsp. *syzygii* in Magelang, Central Java, Indonesia. **(b)** Infected twig. **(c)** Horizontal section of infected twig. **(d)** Bacterial ooze oozing from the infected twig section. Reprinted with permission from Bambang Trianom.

Sumatra disease of clove usually affects productive trees over 10 years of age (Hadiwijaya, 1983). Externally the initial symptom of Sumatra disease of cloves is unseasonal yellowing of leaves followed by leaf-drop from the tips of branches high in the crown (**Figure 2**). However, the leaves may also wilt suddenly and turn brown, but stay attached to the branch. Affected twigs turn reddish brown and progressively die back (**Figures 2a,b**). Symptoms typically progress to lower branches until the whole crown is affected, and the tree dies within 6–18 months (Bennett et al., 1985). Artificial inoculation of *R. syzygii* subsp. *syzygii* on 3 months old *S. aromaticum* seedling leads to symptom appearance beginning with leaf yellowing and drying at 28 days after inoculation and the death of the seedling 56 days after inoculation (Danaatmadja et al., 2009).

Internally, the newly formed wood adjacent to the cambium becomes discolored a pale grayish-brown. When cut infected branches often produce a milky white to pale brown bacterial ooze from the cut surface (**Figure 2c**). The discolouration of the xylem can be traced down the trunk into one or more major roots (Bennett et al., 1985).

Sumatra disease of clove was initially observed in 1975 (Waller and Sitepu, 1975) and further reported in 1985 (Bennett et al., 1985). The disease affects *S. aromaticum* and some species of *Myrtaceae* including some indigenous species in native forests in Indonesia (Lomer et al., 1992), such as *Syzygium aqueum* (Eden–Green et al., 1992). The disease, which was initially confined to the Indonesian provinces of Sumatra and West Java, has now spread to Central Java and East Java and causes economic losses of up to 5–10% per year (Tjahyono, 2013).

Initially Sumatra disease of clove was assumed to be caused by nutritional disorders (Hadiwidjaja, 1956), mineral toxicities (Finck, 1972) as well as disease causing agents such as *Ralstonia solanacearum* [*Xanthomonas solanacearum, Pseudomonas solanacearum* (Hidir, 1973)], *Phytophthora* spp. (Djafaruddin et al., 1979), leaf spot fungi *Phyllostictina* sp. (Kranz, 1976), and the fastidious-xylem limited bacteria (XLB) (Bennett et al., 1985; Hunt et al., 1985) prior to the causative agent being taxonomically described as *Pseudomonas syzygii* (Roberts et al., 1990). Although *R. syzygii* subsp. *indonesiensis* strains have been isolated from the roots and lower trunk of trees only *R. syzygii* subsp. *syzygii* can systemically colonize and kill *S. aromaticum* trees.

*Ralstonia syzygii subsp. syzygii* is included as one of the xylem-restricted or xylem-limited bacteria, which live in xylem cells or tracheary elements of plants (Purcell and Hopkins, 1996). Similar to other diseases caused by xylem-restricted bacteria, *R. syzygii subsp. syzygii* is transmitted by insect vectors that feed on xylem sap (Bové and Garnier, 2002). The tube-building cercopoid (Hemiptera), *Hindola fulva* was found to be the natural insect vector in Sumatra whereas *H. striata* (Hemiptera: Machaerotidae) has been described as the primarily vector in Java (Eden–Green et al., 1992). Vector transmission of *R. syzygii subsp. syzygii* is persistent with a short latent period between acquisition and transmission of the pathogen (Eden–Green et al., 1992). Lomer et al. (1992) suggested that Sumatra disease of cloves may have been transferred to clove trees from an unknown forest hosts because of the localized initial distribution of the disease and the corresponding localized distribution of the vector species. If this hypothesis is correct then the pathogen may have a wider host range than has been previously identified (Purcell and Hopkins, 1996).

Sumatra disease of clove has a distinct pattern of disease expression and distribution in the field. Seedlings less than 2 years old are rarely affected, with trees over 10 years of age being the first to show symptoms and the first to die. The disease advances on a broad front, at an estimated rate of 1–2 km per year and then disappears for years until young trees mature and the cycle repeats (Bennett et al., 1985). As would be expected of an insect transmitted disease the disease spreads in a jump-spread pattern and spreads rapidly in all directions uphill, downhill, and across rivers (Bennett et al., 1985). Bennett et al. (1985) also reported that the rate of spread and symptom expression was partially affected by altitude possibly due to the lower temperature at higher altitudes where rapid decline symptoms are most commonly observed.

### Blood Disease of Banana: *R. syzygii* subsp. *celebesensis*

**FIGURE 3:**
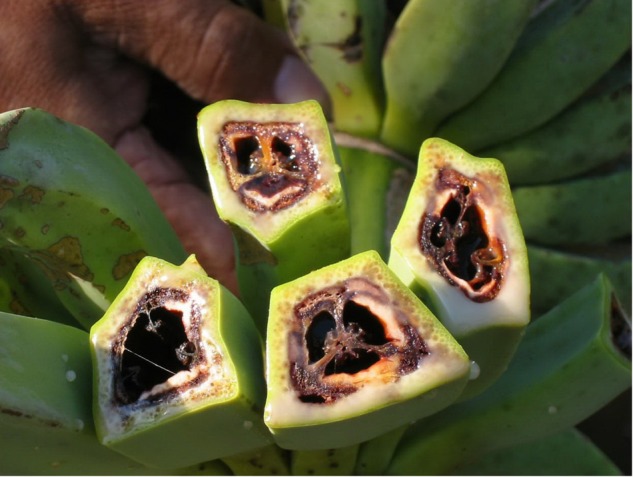
Internal fruit symptoms of blood disease of banana.

Symptoms of Blood Disease are quite similar to Moko disease caused by insect-transmitted strains, the male flower bud and peduncle discolor and shrivel, the fruit pulp shows a reddish dry rot (**Figure 3**), and the vascular tissue throughout the plant exhibits a reddish discoloration, which emits reddish-brown bacterial ooze when cut (Sequeira and Averre, 1961; Buddenhagen, 1962). Moko disease of *Musa* spp. is caused by *R. solanacearum* strains which belong to phylotype II of the *R. solanacearum* species complex (Fegan and Prior, 2006). The older leaves of blood disease-infected *Musa* spp. become yellow, followed by wilting, necrosis and collapse; younger leaves turn bright yellow before becoming necrotic and dry. The pathogen rapidly colonizes the entire plant, and suckers will also wilt and die (Eden-Green, 1994b).

The banana blood disease pathogen is disseminated in soil and water and on farm tools, and has been hypothesized to enter host roots through natural openings or wounds (Gäumann, 1921, 1924). Gäumann (1921) reported that the pathogen survived in soil for at least a year in infested plant residues and infected fruits after entering the plant through its roots. Infested soil, tools, and vehicles move the pathogen within plantations, and movement of infected fruit and planting material enable long-distance spread. Insects that visit *Musa* spp. inflorescences, particularly those of cultivars with dehiscent bracts and an ABB genome (a plantain with one set of chromosomes donated by *Musa acuminata* and two by *Musa balbisiana*), can spread the pathogen rapidly over great distances (Mairawita et al., 2012). *R. syzygii* subsp. *celebesensis* has been isolated from the insect species *Trigona minangkabau* (Mairawita et al., 2012) and *Erionota thrax* (Suharjo et al., 2008). *Erionota thrax* has been observed to visit banana flowers 2–3 times a day (Suharjo et al., 2008). The bracts of ABB/BBB genotype *Musa* spp. are non-persistent and as they fall off they leave abscission scars which provide sites for pathogen entry into the vascular tissue of the plant. Also the male buds of the highly susceptible cultivar “Pisang Kepok” appear to be particularly attractive to insects such as wasps, bees and flies possibly because the male flower nectar has a high sugar content (Setyobudi and Hermanto, 1999).

The transmigration of people from Java to less populated islands in the country appears to be associated with the spread of the disease. *R. syzygii* subsp. *celebesensis* is thought to have originated on Selayar Island near Sulawesi, as the disease was first reported in the early 1900’s after the introduction of dessert bananas (Eden–Green, 1994a). The disease spread to Java in the late 1980’s and has become common on local *M. paradisiaca* cultivars in Sulawesi (Stover and Espinoza, 1992). Unfortunately, the pathogen has spread to most of the larger Indonesian islands, with average yield losses exceeding 35% (Supriadi, 2005), and has also been reported on the island of New Guinea (Davis et al., 2001).

The host range of *R. syzygii* subsp. *celebesensis* is not as wide as the *R. solanacearum* strains causing Moko and Bugtok diseases on *Musa* spp. Baharuddin (1994) showed that *Heliconia* sp. and *Strelitzia reginae*, both relatives of the *Musaceae*, are susceptible to *R. syzygii* subsp. *celebesensis* as are *Canna indica, Datura stramonium, Asclepias currassiva*, and *Solanum nigrum*. Unlike *R. solanacearum* strains causing Moko disease, *R. syzygii* subsp. *celebesensis* is not pathogenic on *S. lycopersicum* and *Solanum melongena* seedlings (Cellier and Prior, 2010; Eden–Green, 1994a; Supriadi, 2005). Strains of *R. syzygii* subsp. *celebesensis* are also not able to infect *Arachis hypogaea, Capsicum* sp., *Nicotiana tabacum, S. tuberosum*, and *Zingiber officinale* (Baharuddin, 1994).

### Bacterial Wilt: *R. syzygii* subsp. *indonesiensis*

**FIGURE 4:**
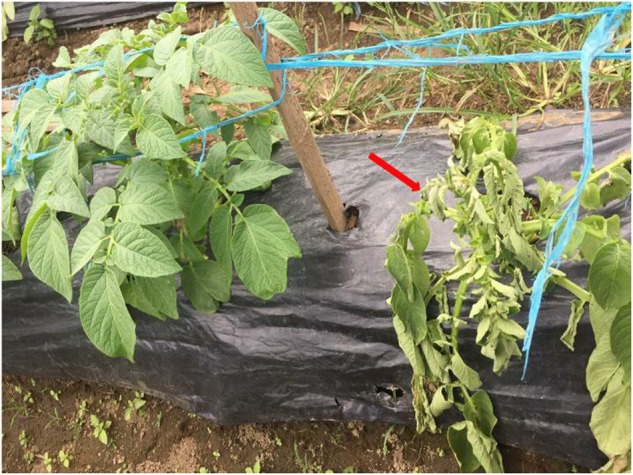
Potato infected by *Ralstonia syzygii* subsp. *indonesiensis* in Magelang, Central Java, Indonesia.

*Ralstonia syzygii* subsp. *indonesiensis* causes disease in a number of solanaceous plants in Indonesia and other countries in Asia but has also has been isolated from clove plants in Indonesia (**Table 1**). The disease symptoms caused by *R. syzygii* subsp. *indonesiensis* strains on solanaceous crops are no different from those described in the past for *R. solanacearum* (Kelman, 1953). The external symptoms of the infected plants are wilting, stunting and yellowing of the foliage with the disease progressing until the plant completely collapses from wilt (**Figure 4**). Internally the vascular tissue becomes progressively discolored in the early stages of infection with portions of the pith and cortex becoming involved as disease develops until complete necrosis occurs.

In Japan *R. syzygii* subsp. *indonesiensis* strains have only been isolated from *S. tuberosum* (Horita et al., 2014). The *R. syzygii* subsp. *indonesiensis* strains isolated from *S. tuberosum* in Japan have been reported to show varying levels of pathogenicity for *S. lycopersicum, A. hypogaea* and *N. tabacum* (Suga et al., 2013). However, none of the strains tested by Suga et al. (2013) were pathogenic for *S. melongena*. A *R. syzygii* subsp. *indonesiensis* strain isolated from *S. lycopersicum* in Indonesia (PSI07) was found to not to cause wilting of *Cucumis melo, Anthurium andraeanum, Musa* spp. and *S. tuberosum* but retained pathogenicity for *S. lycopersicum* (Ailloud et al., 2015).

There is little literature describing the epidemiology and ecology of *R. syzygii* subsp. *indonesiensis* strains. However, as is commonly described for other *R. solanacearum* species complex strains, the bacterium is reported to be able to survive in the field for long periods of time (Suga et al., 2013). Unlike *R. solanacearum* strains which cause brown rot of *S. tuberosum R. syzygii* subsp. *indonesiensis* strains have been shown to only cause disease in *S. tuberosum* in tropical but not temperate conditions (Cellier and Prior, 2010). In comparison to *R. pseudosolanacearum* strains Habe (2016) showed that *R. syzygii* subsp. *indonesiensis* exhibited high pathogenicity for *S. tuberosum* at a lower temperature (26°C) than *R. pseudosolanacearum* strains (28°C).

## Disease Management

### Sumatra Disease of Clove Trees: *R. syzygii* subsp. *syzygii*

All known clove varieties appear equally susceptible (Bennett et al., 1985). As indicated above the spread of the disease in the field is via an insect vector, *Hindola* spp., and most probably contaminated agricultural tools. Therefore local agricultural departments in Indonesia recommend that agricultural tools used for field work should be disinfected between uses, infected plants should be eradicated and insecticide should be applied to minimize the spread of the disease by insect vectors. Although several insecticides have been tested without effective control, aldicarb and carbofuran granules have provided effective control from 7 to 35 and 28 to 217 days after treatment, respectively (Stride and Nurmansyah, 1991). It has further been suggested that if resistant rootstocks could be identified for grafting the degeneration of the roots may be controlled (Bennett et al., 1985). Antibiotics have been shown to be useful in controlling the disease in mature trees for short periods but their use as an effective disease management tool is not recommended (Hunt et al., 1985). Biological control of the pathogen using antagonistic bacterial endophytes and rhizobacteria has also been suggested. Dwimartina et al. (2017) found that endophytic strains of *Bacillus subtilis* subsp. *subtilis* and rhizobacteria which produce indole acetic acid and dissolved phosphate could inhibit the growth of *R. syzygii* subsp. *syzygii*. However, the agropolitical challenges of *S. aromaticum* cultivation in Indonesia has made the application of any disease management strategies difficult (Baharuddin, 1994).

Some natural enemies of *Hindola* spp that may be potential as biological control of the insect vector were identified. Nuhardiyati et al. (1990) reported that *H. fulva* was found in the population with the unknown species of *Hindola, Hindola* sp. in Bengkulu, Sumatra, and Indonesia. *Stylops* sp. was found to parasitize the nymphs and adults of *Hindola* spp. The nymph of family Tettigoniidae was also found and assumed as the predator of Hindola sp. nymphs (Nuhardiyati et al., 1990). On the other hand, Hemipterian parasitoid paratized the nymph and eggs of *Hindola* spp. (Balfas et al., 1990). A member of the genus *Acmopolynema* was the parasite of *Hindola* spp. in Java, Indonesia (Balfas et al., 1990). This parasitoid of the insect vector of *R. syzygii* subsp. *syzygii* is a potential natural biological control agent of the insect vector. Balfas et al. (1990) reported that 30% of *Hindola* spp. eggs collected from clove and 60–80% of eggs collected from the clove related tree, *Xanthostemon chrysanthus*, were parasitized by *Acmopolynema.*

### Blood Disease of Banana: *R. syzygii* subsp. *celebesensis*

It is thought that all edible *Musa* spp. may be susceptible to blood disease as no Indonesian cultivars of *Musa* spp. have been found to be resistant (Gäumann, 1921; Supriadi, 2005). However, some tolerance to blood disease has been reported to occur that may be a source of genetic material for resistance breeding (Supriadi, 2005).

Restricting the movement of planting material from infected areas has been successful in limiting the spread of the disease. A quarantine imposed by the Dutch to limit the spread the disease from Sulawesi was effective for over 60 years until the disease eventually spread to Java around 1987. From this point onward the pathogen has spread rapidly over the Indonesian archipelago and more recently Malaysia.

Removal of the male flower has been found to be effective in controlling the spread of disease as has the use of cultivars that abort the male bud (Hermanto et al., 2013). The cultivar “Pisang Puju,” an acceptable resistant *Musa paradisiaca* variety from Sulawesi, and “Pisang Sepatu Amora” may be suitable because these cultivars abort the male bud, blocking insect transmission (Hermanto et al., 2013).

A combination of basic quarantine and sanitation practices has been suggested to reduce the spread of blood disease (Davis et al., 2001). These measures include prohibiting movement of *Musa* spp. plants or plant parts out of infected areas, using disease free-planting materials, removing male buds immediately after the emergence of the last fruit, pesticide application as soon as the symptoms appear to reduce vector related spread and sterilizing the knives for harvesting.

Biological control of blood disease was suppressed by the application of endophytic bacteria including *Bacillus* sp and *Bacillus subtilis* isolated from *Musa troglodytarum* (Hadiba et al., 2010; Laturapeissa et al., 2014). Since the disease is insect-spread through the bacterial contaminated body of insects visiting *Musa* spp., therefore the biological control of insects associated with *Musa* spp. is potential for limiting the rate of blood disease spread. *Cosmopolites sordidus*, the *Musa* spp. and plantain root and corn borer insect, is not only potential in damaging banana plantation, but also increases the spread rate of blood disease (Subandiyah et al., 2005). This insect pest was reported to be effectively controlled by the application of Steinernematid nematodes (Figueroa, 1988) and *Beauveria bassiana* (Fancelli et al., 2013).

### Bacterial Wilt: *R. syzygii* subsp. *indonesiensis*

Habe (2016) identified that there is a degree of resistance in *S. tuberosum* cultivars to *R. syzygii* subsp. *indonesiensis*. Suga et al. (2013) assessed pathogenic differences between *R. pseudosolanacearum* and *R. syzygii* subsp. *indonesiensis* strains against several Japanese varieties of *S. tuberosum* and breeding lines and indicated that *R. syzygii* subsp. *indonesiensis* strains show high virulence to the breeding lines carrying bacterial wilt resistance conferred from the wild *Solanum* sp., *Solanum phureja*, which is resistant to *R. pseudosolanacearum* strains. Several wild species of *S. tuberosum* such as *S. phureja, Solanum stenostomum*, and *Solanum commersonii* have been used as genetic resources to breed for resistance to bacterial wilt worldwide, and certain new *S. tuberosum* varieties with a high level of resistance have been identified. However, the high levels of resistance of these new *S. tuberosum* varieties have only been confirmed against *R. pseudosolanacearum* or *R. solanacearum* strains. *R. syzygii* subsp. *indonesiensis* strains have not been commonly used as targets in breeding for bacterial wilt resistance, most probably because this organism has only recently been identified as a taxonomic group within the *R. solanacearum* species complex and the restricted distribution [Japan, Korea, the Philippines, India, Indonesia, and Australia (Arwiyanto et al., 2015)] of this organism. Investigations to determine the pathogenicity of *R. syzygii* subsp. *indonesiensis* strains toward for new *S. tuberosum* varieties are needed to address the issue of variability in pathogenicity among different strains. Furthermore, the identification of new genetic resources for breeding need to consider resistance to *R. syzygii* subsp. *indonesiensis* strains in the future.

## Conclusion

*Ralstonia syzygii* with its three subspecies, *R. syzygii* subsp. *syzygii, R. syzygii* subsp. *celebesensis*, and *R. syzygii* subsp. *indonesiensis*, is a phenotypically, genotypically and pathogenically diverse member of the *R. solanacearum* species complex. The members of this species have devastated agricultural commodities including *S. aromaticum, Musa* spp., and solanaceous vegetables in Indonesia. As the diseases caused by these pathogens continue to constrain agricultural production, effective disease management strategies are required.

While *R. syzygii* subsp. *syzygii*, which causes Sumatra disease of cloves, and *R. syzygii* subsp. *celebesensis*, the causal pathogen of banana blood disease, have restricted host ranges and geographic distribution, *R. syzygii* subsp. *indonesiensis* affects many solanaceous crops in several countries in Asia.

Information related to the epidemiology and ecology of *R. syzygii* subsp. *indonesiensis* is limited although it is assumed to behave similarly to *R. solanacearum* and *R. pseudosolanacearum*. Several disease management strategies have been developed and deployed to exclude, prevent and eliminate the pathogen. However, further work is required to confirm the efficacy of current control strategies and to improve implementation to achieve sustainable disease management solutions.

Both *R. syzygii* subsp. *syzygii* and *R. syzygii* subsp. *celebesensis* are insect transmitted pathogens. The dissemination of *R. syzygii* subsp. *syzygii* depends primarily on transmission via the insect vectors *H. fulva* and *H. striata* that feed on xylem sap while *R. syzygii* subsp. *celebesensis* can be disseminated non-specifically by insects visiting *Musa* spp. male buds of infected plants. While control of the diseases caused by *R. syzygii* subsp. *syzygii* and *R. syzygii* subsp. *celebesensis* is possible by the use of insecticides this approach is not widely used in Indonesia. Both *R. syzygii* subsp. *celebesensis* and *R. syzygii* subsp. *syzygii* can also spread through contaminated farm tools, plant material, and other human activities.

As bacterial wilt pathogens are also soil-borne they are difficult to control and successful management usually depends on the eradication and sanitation practices. The application of biosafety practices on infected farms is highly recommended for the management of bacterial wilt diseases. In the face of the high demand of clove for national cigarette production the Indonesian government has preferred to import clove from other countries. This policy has adversely affected the industry and the management of Sumatra disease of clove tree has been hindered because the Indonesian policy does not prioritize the expansion of domestic clove plantations. For blood disease of banana, preventing the spread of the disease by prohibiting the movement of *Musa* spp. plants or plant parts out of infected areas has not been applied effectively in Indonesia due to the difficulties in enforcing quarantine restrictions. The lack of success in the management of these bacterial diseases should become a lesson that promotes the improvement of future control strategies for these important plant diseases.

## Author Contributions

IS and MF: study conception and design of the work. IS, SS, and MF: analysis and interpretation of data. IS and MF: drafting of manuscript and revising it critically for important intellectual content. IS, SS, and MF: final approval of the version to be published. IS, SS, and MF: agreement to be accountable for all aspects of the work in ensuring that questions related to the accuracy or integrity of any part of the work are appropriately investigated and resolved.

## Conflict of Interest Statement

The authors declare that the research was conducted in the absence of any commercial or financial relationships that could be construed as a potential conflict of interest.
